# Correction: Facile preparation of bithiazole-based material for inkjet printed light-emitting electrochemical cell

**DOI:** 10.1039/c9ra90049g

**Published:** 2019-06-18

**Authors:** Jingpei Huo, Wanying Zou, Yubang Zhang, Weilan Chen, Xiaohong Hu, Qianjun Deng, Dongchu Chen

**Affiliations:** Electrochemical Corrosion Institute, College of Materials Science and Energy Engineering, Foshan University Foshan 528000 People’s Republic of China johnhome222@163.com

## Abstract

Correction for ‘Facile preparation of bithiazole-based material for inkjet printed light-emitting electrochemical cell’ by Jingpei Huo *et al.*, *RSC Adv.*, 2019, **9**, 6163–6168.

The authors regret that the funding information in the acknowledgements of the original article was incorrect. “Guangdong Natural Science Foundation of China (Grant No. 2018A030310350)” should be “Guangdong Natural Science Foundation of China (Grant No. 2018A1660001)”.

The Royal Society of Chemistry apologises for these errors and any consequent inconvenience to authors and readers.

## Supplementary Material

